# Tropism, Cytotoxicity, and Inflammatory Properties of Two Envelope Genes of Murine Leukemia Virus Type-Endogenous Retroviruses of C57BL/6J Mice

**DOI:** 10.1155/2011/509604

**Published:** 2011-06-05

**Authors:** Young-Kwan Lee, Alex Chew, David G. Greenhalgh, Kiho Cho

**Affiliations:** Shriners Hospitals for Children Northern California and Department of Surgery, University of California, Davis, 2425 Stockton Boulevard, Sacramento, CA 95817, USA

## Abstract

Envelope (*env*) proteins of certain endogenous retroviruses (ERVs) participate in various pathophysiological processes. In this study, we characterized pathophysiologic properties of two murine leukemia virus-type ERV (MuLV-ERV) *env* genes cloned from the ovary of C57BL/6J mice. The two *env* genes (named ENV_OV1_ and ENV_OV2_), with 1,926 bp coding region, originated from two MuLV-ERV loci on chromosomes 8 and 18, respectively. ENV_OV1_ and ENV_OV2_ were ~75 kDa and predominantly expressed on the cell membrane. They were capable of producing pseudotype murine leukemia virus virions. Tropism trait and infectivity of ENV_OV2_ were similar to the polytropic *env*; however, ENV_OV1_ had very low level of infectivity. Overexpression of ENV_OV2_, but not ENV_OV1_, exerted cytotoxic effects and induced expression of COX-2, IL-1*β*, IL-6, and iNOS. These findings suggest that the ENV_OV1_ and ENV_OV2_ are capable of serving as an *env* protein for virion assembly, and they exert differential cytotoxicity and modulation of inflammatory mediators.

## 1. Introduction

Ancient infection of germline cells with exogenous retroviruses established a genome-wide random embedment of proviruses, called endogenous retroviruses (ERVs), and Mendelian genetics governs their inheritance to the offsprings [1]. ERVs are reported to exist in the genome of all vertebrates and constitute approximately 8% of the human genome and 10% of the mouse genome [2–4]. The majority of ERVs identified so far are reported to be defective primarily based on their inability to encode intact polypeptides for *gag* (group specific antigen), *pol* (reverse transcriptase), and *env* (envelope) genes, which are essential for the retroviral life cycle [5]. However, recent studies identified a number of ERVs, which retain intact coding potentials for *gag*, *pol*, and/or *env* genes, and some of them are reported to be associated with a range of normal physiology (e.g., placental morphogenesis) as well as pathogenic processes (e.g., multiple sclerosis, schizophrenia, injury, and chronic fatigue syndrome) [6–10]. On the other hand, biology of porcine ERVs (PERVs) has been studied extensively because of the potential transmission of PERVs to humans as an adverse side effect of xenotransplantation [11]. 

The *env* glycoproteins of certain human ERVs (HERVs) have been implicated in diverse disease processes [12–16]. For instance, the *env* glycoproteins of HERV-K, HERV-E, and ERV-3 were characterized as tumor-associated antigens in different types of cancer [15–18]. The HERV-W *env* glycoprotein, called syncytin-1, is highly expressed in glial cells within central nervous system of multiple sclerosis, an autoimmune disease, patients [13]. It is proposed that potent proinflammatory properties of syncytin-1 contribute to neuronal inflammation and resultant damage to oligodendrocytes during the progression of multiple sclerosis [12]. On the other hand, syncytin-1 and HERV-FRD *env* glycoprotein, called syncytin-2, are reported to play an essential role during embryonic development by controlling formation of placental syncytiotrophoblasts primarily through their highly fusogenic properties [19–22]. Additional *env* glycoproteins have been identified and characterized from murine ERVs (syncytin-A and syncytin-B) and endogenous Jaagsiekte sheep retrovirus (enJSRV), and their roles in placenta morphogenesis are similar to syncytin-1 and syncytin-2 [7, 23, 24]. The findings from recent studies provide evidence suggesting that *env* glycoproteins of certain ERVs play a critical role in biological processes of normal physiology as well as diseases. 

During a survey of expression profile of MuLV-ERV subgenomic *env* transcripts in various normal tissues of C57BL/6J mice, two putative full-length *env* transcripts were identified in the ovary. In this study, the biological characteristics of these two MuLV-ERV *env* genes, named ENV_OV1_ and ENV_OV2_, were investigated by examining a selective set of pathophysiologic parameters.

## 2. Materials and Methods

### 2.1. Animals

Female C57BL/6J mice (approximately 12 weeks old) were purchased from the Jackson Laboratory (Bar Harbor, Me) and housed according to the guidelines of the National Institutes of Health. The Animal Use and Care Administrative Advisory Committee of the University of California, Davis, approved the experimental protocol. Three mice were sacrificed by cervical dislocation for tissue collection without any pretreatment, and tissue samples were snap-frozen.

### 2.2. RT-PCR Analyses

RNA isolation and cDNA synthesis were performed primarily according to the relevant protocols provided by the kit manufacturer. Briefly, total RNAs were extracted using an RNeasy kit (Qiagen, Valencia, Calif) and cDNAs were synthesized using 100 ng of total RNA from each sample (tissue or cell) and the Sensiscript reverse transcriptase (Qiagen). The primers capable of amplifying the full length as well as subgenomic MuLV-ERV transcripts were designed based on the MAIDS (murine acquired immunodeficiency virus) virus-related provirus (GenBank No. S80082) [25]: forward, 5′-CAT TTG GAG GTC CCA CCG AGA-3′ (MV1K) and reverse, 5′-CTC AGT CTG TCG GAG GAC TG-3′ (MV2D). The following are the primer sets used for inflammatory mediators: COX-2 (forward, 5′-ACA CAG TGC ACT ACA TCC TGA C-3′ and reverse, 5′-ATC ATC TCT ACC TGA GTG TC-3′), ICAM-1 (forward, 5′-AGC TGT TTG AGC TGA GCG AGA-3′ and reverse, 5′-CTG TCG AAC TCC TCA GTC A-3′), IL-1*β* (forward, 5′-GAC AGT GAT GAG AAT GAC CTG-3′ and reverse, 5′-GAA CTC TGC AGA CTC AAA CTC CA-3′), IL-6 (forward, 5′-GCC TTC CCT ACT TCA CAA GTC CG-3′ and reverse, 5′-CAC TAG GTT TGC CGA GTA GAT CTC-3′) [26], iNOS (forward, 5′-ACA AGC TGC ATG TGA CAT CGA-3′ and reverse, 5′-CAG AGC CTG AAG TCA TGT TTG C-3′), and TNF-*α* (forward, 5′-GCA TGA TCC GCG ACG TGG AA-3′ and reverse, 5′-AGA TCC ATG CCG TTG GCC AG-3′) [27]. In addition, *β*-actin (forward, 5′-CCA ACT GGG ACG ACA TGG AG-3′ and reverse, 5′-GTA GAT GGG CAC AGT GTG GG-3′) was used as an internal expression control [28]. The density of amplified products (applied only for inflammatory mediators) was measured using KODAK Molecular Imaging Software ver. 4.5 (Carestream Health, Rochester, NY), and it was normalized to *β*-actin control.

### 2.3. Cloning and Sequencing of env Transcripts

The RT-PCR products of the MuLV-ERV subgenomic transcripts (~2.9 Kb) were cloned into the pGEM-T Easy vector (Promega, Madison, Wis) followed by plasmid DNA preparation using a kit from Qiagen, and sequencing analysis at Davis Sequencing Inc (Davis, Calif) or Molecular Cloning Laboratory (South San Francisco, Calif). DNA sequences were analyzed using Vector NTI-ver. 10 (Invitrogen, Carlsbad, Calif) or Editseq and MegAlign program within DNASTAR ver. 8.0.2 (DNASTAR, Madison, Wis).

### 2.4. Construction of ENV_OV1_ and ENV_OV2_ Expression Vectors

The coding regions of the ENV_OV1_ and ENV_OV2_ were amplified by PCR from their respective original cDNA clones using a set of primers embedded with restriction enzyme sites for cloning into the pcDNA4/HisMax (Invitrogen): forward with *Not*I, 5′-CGC GGC GGC CGC ATG GAA GGT CCA GCG TTC TC-3′, ENV_OV1_-reverse with *Xho*I, 5′-GGC TCG AGT TAT TCA CGT GAT TCC ACT TTT TCT GG-3′, and ENV_OV2_-reverse with *Xho*I, 5′-GGC TCG AGT TAT TCA CGT GAT TCC ACT TCT TCT GG-3′. The amplified coding sequences after 10 PCR cycles were cloned into the pGEMT-Easy vector (Promega) followed by digestion with *Not*I and *Xho*I and subsequently cloned into pcDNA4/HisMax (Invitrogen).

### 2.5. Cell Lines

The GP2-293 packaging cells (purchased from Clontech, Mountain View, Calif), tsA201 cells (a derivative of HEK293 cells), COS-7 cells, and COS-1 cells were maintained in Dulbecco's modified eagle medium (DMEM, Invitrogen) supplemented with 10% fetal bovine serum, streptomycin, and penicillin G. Five other cell lines (HeLa, Neuro-2a, MDCK, HCT 116, and NIH3T3) were cultured according to the protocols recommended by the American Type Culture Collection (Manassas, Va).

### 2.6. Assays for Production, Tropism, and Infectivity of Pseudotype LacZ-MuLV Virions

The GP2-293 cells, which were seeded onto a 6-well plate at a concentration of 5 × 10^5^ cells per well, were cotransfected with pQCLIN (Clontech, Mountain View, Calif) and pcDNA4/HisMax-ENV_OV1_ or pcDNA4/HisMax-ENV_OV2_ plasmid using Lipofectamine 2000 (Invitrogen). The following *env* proteins were used for tropism and infectivity controls: ecotropic (pEco), 4070A amphotropic (pAmpho), 10A1 amphotropic (p10A1) with a broader host range than 4070A, and G glycoprotein of the vesicular stomatitis virus (VSV-G) (Clontech). Culture supernatants containing pseudotype viral particles were passed through a 0.45 *μ*M filter (Fisher Scientific, Pittsburgh, Pa). Transfection efficiency was estimated by counting the stained cells under the microscope after X-gal staining.

For each cell line (a total of 8 cell lines) employed for tropism and infection analysis, 5 × 10^4^ cells/well were seeded onto a 24-well plate and incubated overnight in preparation of viral transduction. Subsequently, the medium was replaced with 0.5 mL of serial dilutions of culture supernatants containing pseudotype LacZ-MuLV virions, in which Polybrene (Sigma, Milwaukee, Wis) was added (8 *μ*g/mL), followed by washing after 4 hours and incubation with 0.5 mL of fresh media for 2 days. The infected cells were treated with fixing solution (2% formaldehyde and 0.2% glutaraldehyde in PBS) and stained with X-gal solution. Cells stained blue were counted under the microscope as an infection unit.

### 2.7. Western Blot Analyses

To confirm expression of the pcDNA4/HisMax-ENV_OV1_ or pcDNA4/HisMax-ENV_OV2_ construct, Western blot analysis was performed following transfection into tsA201 cells using Fugene 6 reagent (Roche, Mannheim, Germany). At 2 days after transfection, the cells were harvested, and Western blot analysis was performed. Briefly, the membrane, blocked in 5% nonfat dry milk (NFDM), was incubated with a goat antibody specific for gp69/71 of Rauscher MuLV (1 : 2000 dilution with 5% NFDM in TBST [Tris-buffered saline with Tween 20]) obtained from ViroMed Biosafety Laboratories (Camden, NJ) followed by an anti-goat-HRP antibody (1 : 5000, Santa Cruz Biotechnology, Santa Cruz, Calif). The protein signal was visualized using ECL reagents (GE healthcare, Pittsburgh, Pa). A similar protocol was used to detect *env* glycoprotein from supernatants of the GP2-293 cells producing pseudotype LacZ-MuLV virions.

### 2.8. Immunocytochemistry

HeLa cells, which were transfected with the pcDNA4/HisMax-ENV_OV1_ or pcDNA4/HisMax-ENV_OV2_ construct, were harvested and transferred into 0.1% poly-L-Lysine coated coverslips and incubated for 1 day. Cells were then immunostained with a goat antibody specific for gp69/71 (1 : 200 diluted in culture medium, ViroMed Biosafety Laboratories) and fixed with both 4% paraformaldehyde. Fixed cells were incubated with a Texas-Red-conjugated antigoat IgG secondary antibody (1 : 200 diluted in PBS, Vector Laboratories, Burlingame, Calif) and stained cells were visualized by a Zeiss microscope using AxioVison software version 4.5 (Carl Zeiss, Jena, Germany).

### 2.9. Cytotoxicity and Cell Proliferation Assays

HeLa cells, which were transfected with the pcDNA4/HisMax-ENV_OV1_ or pcDNA4/HisMax-ENV_OV2_ construct, were subjected to cytotoxicity assay using a Cytotoxicity Detection Kit (Roche, South San Francisco, Calif) according to the protocol recommended by the manufacturer. Absorbance was measured at 490 nm with a reference at 600 nm using a reader from Molecular Devices (Sunnyvale, Calif). Cell proliferation rate was measured from these cells using the colorimetric MTT (3- (4, 5-dimethylthiazol-2-yl)-2, 5-diphenyltetrazolium bromide) (Sigma, Milwaukee, Wis) assay as described previously [29]. Absorbance was read at 560 nm with a reference at 600 nm using a reader (Molecular Devices). All experiments were performed at least in triplicate, and 4 independent experiments were repeated.

### 2.10. Analysis of Inflammatory Mediators

RAW264.7 cells, which were transfected with the pcDNA4/HisMax-ENV_OV1_ or pcDNA4/HisMax-ENV_OV2_ construct, were harvested at 1 day after transfection, and they were examined for expression of a set of inflammatory mediators at mRNA levels by RT-PCR, and the relevant protocols and reagents are described in Section 2.2 above.

### 2.11. Statistical Analysis

Statistical analysis was performed using two-tailed Student's *t*-test and statistical significance was determined as *P < .05 and **P < .01.

## 3. Results

### 3.1. Identification and Initial Characterization of Two MuLV-ERV env Subgenomic Transcripts Expressed in the Ovary of C57BL/6J Mice

**Figure 1:**
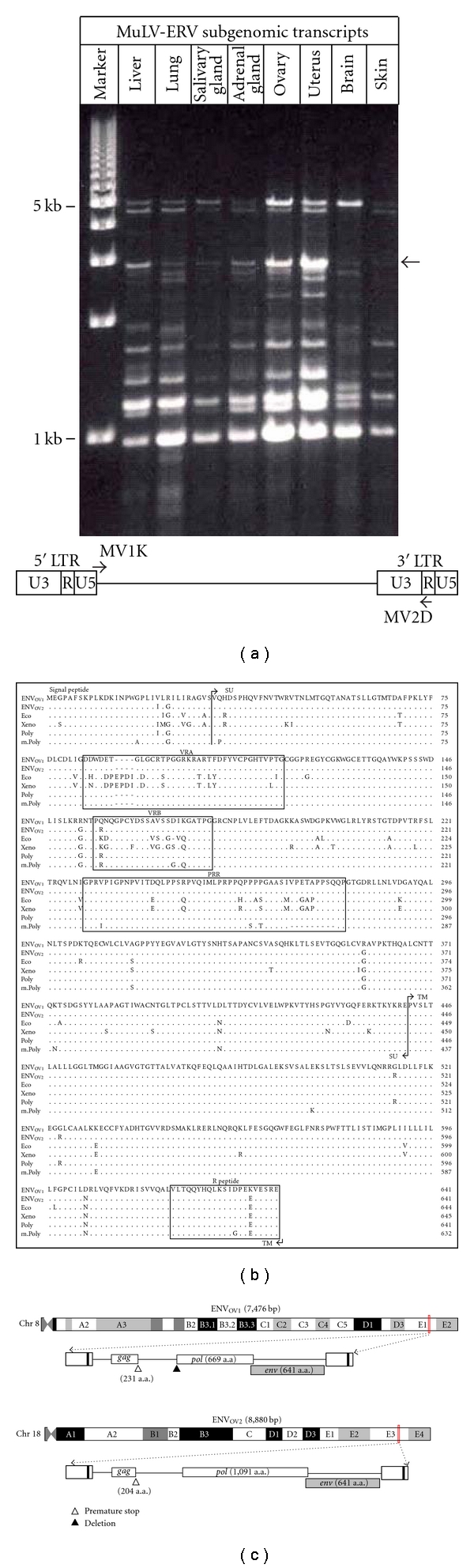
Identification of full-length *env* transcripts from the ovary of C57BL/6J mice. (a) A number of MuLV-ERV subgenomic transcripts were expressed in normal tissues (liver, lung, salivary gland, adrenal gland, brain, and skin) of C57BL/6J mice. A schematic diagram indicates the locations of primers used for amplification of the subgenomic transcripts. (b) The amino acid sequences of two intact *env* genes, named ENV_OV1_ and ENV_OV2_, which were isolated from the ovary (indicated with an arrow in panel (a)), were compared to reference *env* polypeptides with known tropism traits (GeneBank accession number: AAG39911 (Eco), ACY30460 (Xeno), AAO37283 (Poly), and AAA88318 (m.Poly)). (c) The putative MuLV-ERV proviruses harboring the ENV_OV1_ and ENV_OV2_ genes were mapped to chromosomes 8 and 18 of C57BL/6J genome, respectively. SU (surface domain), TM (transmembrane domain), VRA (variable region A), VRB (variable region B), PRR (proline rich region), LTR (long terminal repeat), R (repeat), and U (unique region).

The expression profiles of MuLV-ERV *env* genes in various normal tissues (liver, lung, salivary gland, adrenal gland, brain, skin, ovary, and uterus) of C57BL/6J mice were investigated. A number of putative subgenomic transcripts with varying sizes, ranging from ~1 Kb to ~5 Kb, which may be generated by splicing and/or deletion, were differentially expressed in each tissue. Among them were ~2.9 Kb bands presumed to be amplified from full-length MuLV-ERV *env* transcripts, and their expression was evident in the ovary and uterus as well as other tissues (Figure 1(a)). Sequencing analysis revealed that the two 2,892 bp transcripts were *env* mRNAs, which were generated by a single splicing using the well-characterized donor and acceptor signals [30]. A subsequent open reading frame analysis revealed that the two full-length MuLV-ERV *env* genes, named ENV_OV1_ and ENV_OV2_, retain intact coding potential for *env *glycoproteins of 641 amino acids. While the nucleotide and polypeptide sequences of the ENV_OV2_ was identical to an *env* gene of an polytropic murine leukemia virus (MuLV)-related retroviral sequence from NFS/N mice, the ENV_OV1_ has not been reported yet [31].

Prior to the functional characterization of the ENV_OV1_ and ENV_OV2_, they were aligned with four different reference *env* polypeptides displaying different host tropisms: ecotropic, xenotropic, polytropic, and modified polytropic (Figure 1(b)). It turned out that both ENV_OV1_ and ENV_OV2_ had a higher level of sequence similarity to the polytropic/modified polytropic *env* polypeptides compared to the others. Both the ENV_OV1_ and ENV_OV2_ share the identical sequence in the variable region A and proline rich region, while one amino acid residue was different in the variable region B and R peptide, respectively. To identify the putative MuLV-ERVs encoding the ENV_OV1_ and ENV_OV2_, respectively, the C57BL/6J genome sequence (Build 37.1) from the National Center for Biotechnology Information (NCBI) was surveyed with the respective *env* nucleotide sequences using the BLAST program [32]. The putative proviruses presumed to encode the ENV_OV1_ and ENV_OV2_ were mapped to ideogram data of chromosome 8 and ideogram data of chromosome 18, respectively. Both MuLV-ERVs retained the coding potential for *env* polypeptide, and ENV_OV2_ also had the coding potential for *pol* polypeptide (Figure 1(c)). 

**Figure 2:**
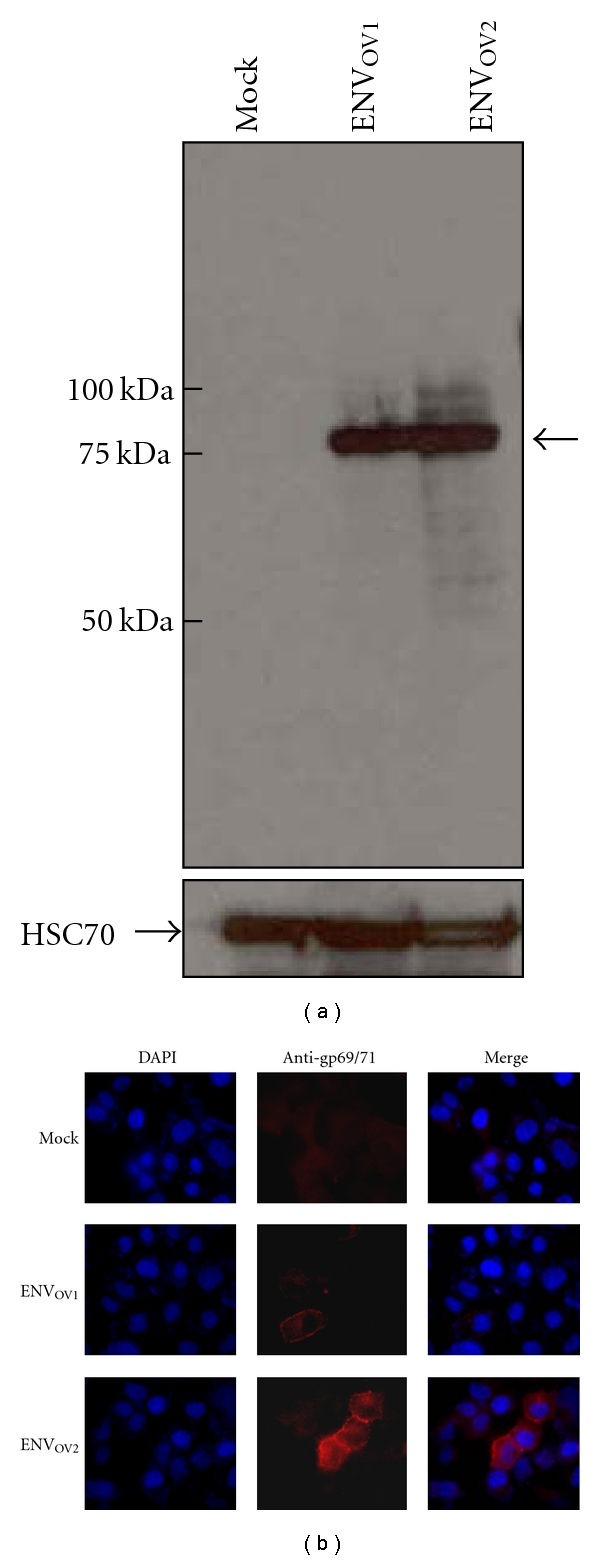
Coding potential and membrane localization of ENV_OV1_ and ENV_OV2_ polypeptides. (a) The coding potential of the ENV_OV1_ and ENV_OV2_ polypeptides was confirmed by overexpression followed by Western blot analysis using antibody against Rauscher MuLV gp69/71 *env* polypeptide. (b) The cellular distribution of the overexpressed ENV_OV1_ and ENV_OV2_ polypeptides was examined by immunocytochemistry using antibody against Rauscher MuLV gp69/71 polypeptide, and their membrane staining pattern was evident. The cells transfected with a blank plasmid serves as a negative control (mock). DAPI (4′,6-diamidino-2-phenylindole).

To examine whether the ENV_OV1_ and ENV_OV2_ are able to produce full-length *env* polypeptides, they were overexpressed in a human cell line followed by Western blot detection using an anti-gp69/71 (*env*) antibody. A protein band of ~75 kDa, which was about the size of MuLV-ERV *env* polypeptides, was detected (Figure 2(a)). Moreover, the subcellular distribution of these *env* polypeptides was examined by transient transfection followed by immunocytochemistry using the same antibody used for the Western blot analysis. Both the ENV_OV1_ and ENV_OV2_ proteins were evidently expressed on the cell membrane as was expected from the retroviral *env* polypeptides (Figure 2(b)).

### 3.2. Infectivity and Tropism Traits of ENV_OV1_ and ENV_OV2_ Polypeptides

**Figure 3:**
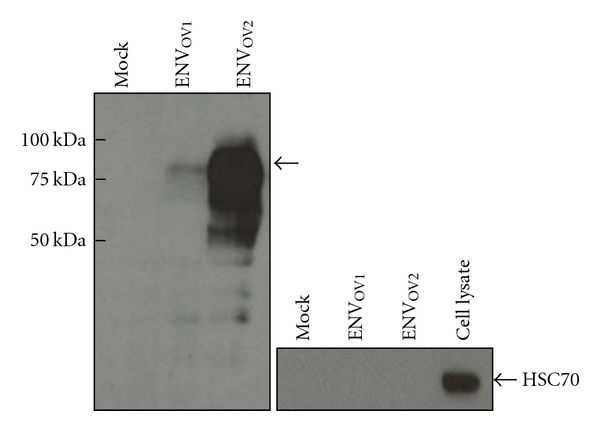
Production of pseudotype LacZ-MuLV virions. Presence of the pseudotype LacZ-MuLV virus particles in culture supernatants of the GP2-293 packaging cells was confirmed by detection of the *env* polypeptides using antibody against Rauscher MuLV gp69/71. Arrow indicates the *env* polypeptides. Supernatants collected from cells transfected with a blank plasmid serves as a negative control (mock).

Two relevant characteristics, tropism and infectivity, of the ENV_OV1_ and ENV_OV2_ polypeptides were determined using a retroviral packaging system and compared to reference *env* proteins with known host tropisms: ecotropic, amphotropic, and pantropic. Prior to the analyses of infectivity and tropism traits, the packaging potential of the ENV_OV1_ and ENV_OV2_ polypeptides and release of pseudotype LacZ-MuLV virions were confirmed by detection of ~75 kDa bands in the culture supernatants collected after transfection (Figure 3). Interestingly, a markedly higher level of *env* protein was detected in the supernatants presumed to contain ENV_OV2_-packaged pseudotype virions compared to the ENV_OV1_ samples. This finding may suggest that the ENV_OV2_ polypeptide is more efficiently produced and/or packaged during the course of virion assembly compared to the ENV_OV1_. The infectivity and tropism traits of the ENV_OV1_ and ENV_OV2_ polypeptides were then examined by infection of various cell types derived from human, nonhuman primate, mouse, and dog. It revealed that the pseudotype LacZ-MuLV virions packaged with either ENV_OV1_ or ENV_OV2_ were capable of infecting both mouse as well as nonmouse cells suggesting their polytropic tropism trait, which is consistent with the alignment data presented in Figure 1 (Table 1). While the pseudotype ENV_OV2_-LacZ-MuLV virions demonstrated infectivity that is very similar to the amphotropic and pantropic controls, the ENV_OV1_-LacZ-MuLV virions had substantially low infection titers compared to the controls, probably due to low expression level and/or inefficient packaging potential during virion assembly.

### 3.3. Cytopathic Characteristics of ENV_OV1_ and ENV_OV2_ Polypeptides

**Figure 4:**
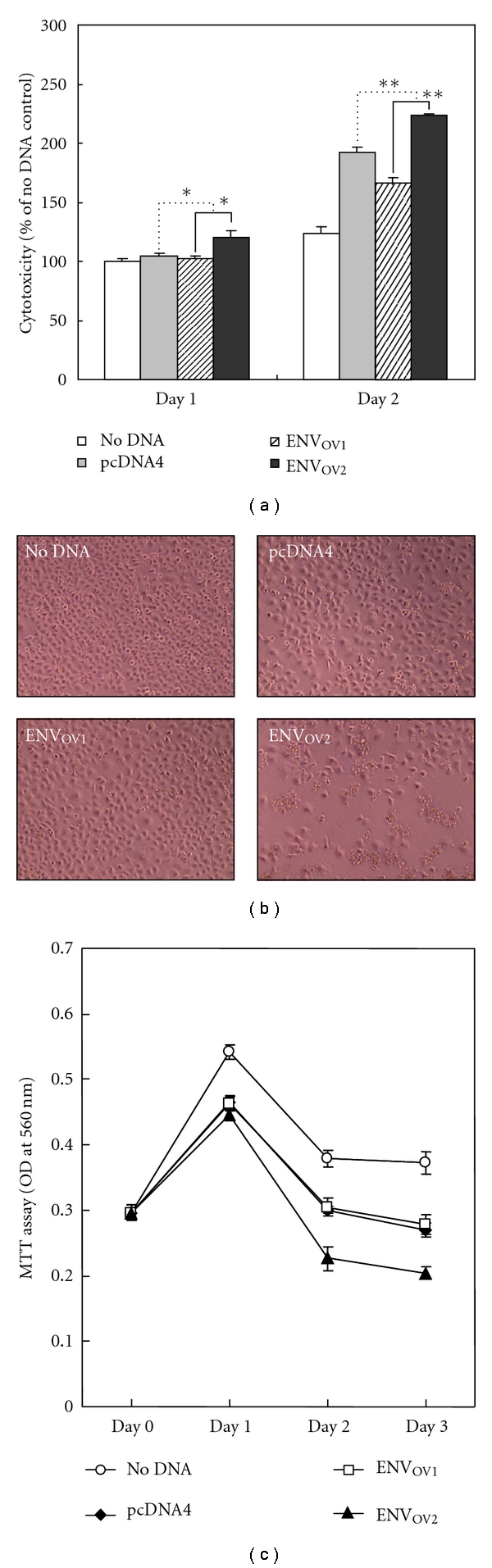
Cytopathic effects of the ENV_OV1_ and ENV_OV2_ polypeptides. (a) and (b) Cytotoxic property of the ENV_OV2_ polypeptide, but not ENV_OV1_ polypeptide, was observed during cytotoxicity assay by measurement of lactate dehydrogenase release and morphologic examination of cells (200x magnification). The degree of cytotoxicity was normalized to the no DNA (Panel (a)). (c) ENV_OV2_ polypeptide's inhibitory effect on cell proliferation was demonstrated by MTT assay, which measures cell viability, following overexpression. All experiments were performed in triplicate. ∗ and ∗∗ indicate statistical significance (*P < .05; **P < .01).

In this experiment, the cytopathic effects of the ENV_OV1_ and ENV_OV2_ polypeptides were examined by overexpression followed by measurement of cytotoxicity and inhibition of cell proliferation. Cytotoxic property of the ENV_OV2_ polypeptide was clearly demonstrated by both colorimetric quantitative assay and microscopic examination of morphological characteristics, including adherence to culture plate (Figures 4(a) and 4(b)). In contrast, no significant cytotoxic effects were observed in the cells overexpressed with the ENV_OV1_ polypeptide compared to negative controls. On the other hand, the overexpression of the ENV_OV2_ polypeptide, but not ENV_OV1_ polypeptide, evidently inhibited cell proliferation, which was measured by colorimetric quantitation of cell growth (Figure 4(c)). It is likely that inhibition of cell proliferation by the ENV_OV2_ polypeptide is linked to its cytotoxic effect, and it is unclear how its high infection titer correlates with the cytopathic characteristics.

### 3.4. Modulation of Inflammatory Mediators by ENV_OV1_ and ENV_OV2_ Polypeptides

**Figure 5:**
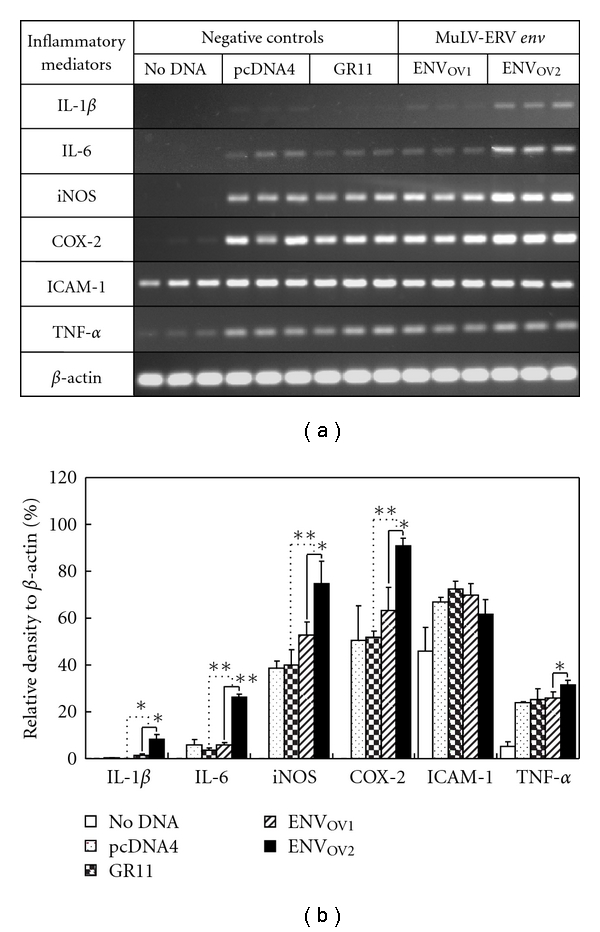
Effects of ENV_OV1_ and ENV_OV2_ polypeptides on expression of inflammatory mediators. (a) and (b) The effects of the ENV_OV1_ or ENV_OV2_ polypeptide in RAW264.7 on the expression of various inflammatory mediators are presented. Differential modulation potentials for inflammatory mediators were observed between ENV_OV1_ and ENV_OV2_ polypeptides. The densitometric value of each inflammatory mediator was normalized to *β*-actin, and a graph was formulated. Three different forms of negative control were included in this experiment: no DNA, pcDNA4 (blank pcDNA4/HisMax plasmid), and GR11 (similar insert size as ENV_OV1_ and ENV_OV2_: mouse glucocorticoid receptor in pcDNA4/HisMax). The assay was performed in triplicate. ∗ and ∗∗ indicate statistical significance (*P < .05; **P < .01).

To investigate whether the ENV_OV1_ and ENV_OV2_ play a role in inflammation, changes in mRNA expression of a set of inflammatory mediators were surveyed following their overexpression in RAW264.7 alveolar macrophage cells. The set include proinflammatory mediators of COX-2 (cyclooxygenase-2), ICAM-1 (intercellular adhesion molecule 1), iNOS (inducible nitric oxide synthase), IL-1*β*, IL-6, and TNF-*α*. The expression of four proinflammatory mediators, COX-2, iNOS, IL-1*β*, and IL-6, was significantly increased by overexpression of ENV_OV2_ polypeptide but not ENV_OV1_ (Figures 5(a) and 5(b)). In contrast, no significant changes in ICAM-1 and TNF-*α* levels were detected following the overexpression of either the ENV_OV1_ or the ENV_OV2_ polypeptide. The findings from this study suggest that the ENV_OV1_ and ENV_OV2_ polypeptides differentially participate in certain signaling events controlling the production of inflammatory mediators.

## 4. Discussion

Two MuLV-ERV* env* genes with intact coding potential, named ENV_OV1_ and ENV_OV2_, were isolated from the ovary of normal C57BL/6J mice and their biological properties were characterized. Although the sequence of one (ENV_OV2_) of the two *env* genes has been reported previously, its biological functions have not been characterized [31]. The findings from this study suggest that both the ENV_OV1_ and ENV_OV2_ polypeptides, which were determined to confer polytropic tropism, participate in a range of biological processes, such as retroviral packaging, cell death, proliferation, and inflammation.

The results from this study suggest that putative MuLV-ERVs, or unidentified exogenous retroviruses, which are packaged with either ENV_OV1_ or ENV_OV2_ polypeptide, are capable of infecting cells of mice as well as other species, such as humans, nonhuman primates, and dogs. *De novo* as well as stress-related activation of the MuLV-ERVs, which are packaged with these *env* polypeptides, may be followed by infection of specific cells of local as well as distant. In addition to the potential cytopathic effects examined in this study, the genomic random integration of the proviral DNAs may be directly linked to various pathogenic outcomes following infection. Further *in vivo* studies are needed to determine infectivity of the MuLV-ERVs packaged with these *env* polypeptides in mice as well as other species.

ERVs have been associated with a range of diseases, such as sepsis, multiple sclerosis, and injury whose central pathology includes inflammatory conditions [12, 33–37]. Some reports provided evidence that certain ERV *env* gene products, but not the relevant virus particles, play a role in the inflammatory processes associated with various pathologic phenotypes [38, 39]. The HERV-W syncytin-1 exerted its inflammatory effects by induction of proinflammatory mediators, such as IL-1*β*, IL-6, IL-12, iNOS, and TNF-*α*, leading to neuron inflammation in multiple sclerosis patients [12, 40]. The findings from this study that the ENV_OV2_ polypeptide is capable of modulating inflammatory mediators suggest its potential roles in immunologic homeostasis as well as in various diseases involving inflammatory conditions, such as sepsis [41, 42]. 

A markedly higher level of packaging and subsequent release of pseudotype LacZ-MuLV virions was predicted with the ENV_OV2_ compared to the ENV_OV1_, based on the detection of abundant *env* polypeptide in the culture supernatants of ENV_OV2_ samples. It is consistent with the finding that the ENV_OV2_-packaged virions (pseudotype ENV_OV2_-LacZ-MuLVs) had higher infection titers compared to the ENV_OV1_-packaged virions (pseudotype ENV_OV1_-LacZ-MuLVs). It is possible that the putative high packaging rate with the ENV_OV2_ polypeptide is directly linked to its efficient transcription and/or translation as well as stability. On the other hand, abundant presence of the ENV_OV2_ polypeptide in the cytoplasm may explain, at least in part, the characteristics of cytotoxicity and inhibition of proliferation compared to the ENV_OV1_. Throughout the entire coding sequences, nine amino acid residues (V22I, R24G, R158G, Q161R, R362G, G518R, G528R, D608, and K640E) were different between the ENV_OV1_ and ENV_OV2_ polypeptides. Further investigation may be needed to learn the roles of these polymorphic residues in stability as well as pathogenic characteristics, including infectivity, of the ENV_OV1_ and ENV_OV2_ polypeptides.

## 5. Conclusions

The findings from this study indicate that certain MuLV-ERV *env* polypeptides, such as ENV_OV2_, may participate in a range of pathophysiologic processes as an envelope of MuLV-ERV virions and/or independently.

## Figures and Tables

**Table 1:** Tropism trait and infectivity of the ENV_OV1_ and ENV_OV2_ polypeptides. Infection titer unit (U/mL): number of LacZ positive cells per mL of supernatant containing virus particles. ND: not detectable.

Cell lines			Infection titer (U/mL)			
	ENV_OV1_	ENV_OV2_	pEco	pAmpho	p10A1	pVSVG
Human						
HeLa	1.6 × 10^1^	1.5 × 10^4^	ND	2.2 × 10^4^	2.2 × 10^4^	4.7 × 10^4^
tsA201	3.1 × 10^1^	2.0 × 10^6^	ND	2.8 × 10^6^	1.4 × 10^6^	8 × 10^6^
HCT116	2.0 × 10^1^	3.2 × 10^4^	ND	2.7 × 10^4^	4.0 × 10^4^	1.7 × 10^4^

Nonhuman primate						
COS-1	4.0 × 10^1^	2.0 × 10^5^	ND	2.0 × 10^5^	3.8 × 10^5^	1.2 × 10^5^
COS-7	6.9 × 10^1^	8.4 × 10^5^	ND	1.3 × 10^6^	3.2 × 10^6^	5.2 × 10^6^

Mouse						
NIH3T3	2.0 × 10^1^	9.5 × 10^4^	4.5 × 10^5^	1.7 × 10^4^	3.3 × 10^4^	1.4 × 10^4^
Neuro2a	ND	2.1 × 10^4^	4.4 × 10^3^	2.7 × 10^3^	2.1 × 10^3^	4.8 × 10^3^

Dog						
MDCK	ND	3.2 × 10^1^	ND	3.2 × 10^2^	2.6 × 10^2^	1.2 × 10^2^
